# Muscle activity of upper and lower trapezius and serratus anterior during unloaded and maximal loaded shoulder flexion and extension

**DOI:** 10.1080/23335432.2017.1364668

**Published:** 2017-12-15

**Authors:** Monique Wochatz, Sophie Rabe, Martin Wolter, Tilman Engel, Steffen Mueller, Frank Mayer

**Affiliations:** a University Outpatient Clinic, Sports Medicine & Sports Orthopaedics, University of Potsdam, Potsdam, Germany; b Center of Rehabilitation Research, University of Potsdam, Potsdam, Germany

**Keywords:** Shoulder, scapular muscle activity, isokinetic testing, electromyography

## Abstract

Altered scapular muscle activity is mostly described under unloaded and submaximal loaded conditions in impingement patients. However, there is no clear evidence on muscle activity with respect to movement phases under maximum load in healthy subjects. Therefore, this study aimed to investigate scapular muscle activity under unloaded and maximum loaded isokinetic shoulder flexion and extension in regard to the movement phase. Fourteen adults performed unloaded (continuous passive motion [CPM]) as well as maximum loaded (concentric [CON], eccentric [ECC]) isokinetic shoulder flexion (Flex) and extension (Ext). Simultaneously, scapular muscle activity was measured by EMG. Root mean square was calculated for the whole ROM and four movement phases. Data were analyzed descriptively and by two-way repeated measures ANOVA. CPM_Flex_ resulted in a linear increase of muscle activity for all muscles. Muscle activity during CON_Flex_ and ECC_Flex_ resulted in either constant activity levels or in an initial increase followed by a plateau in the second half of movement. CPM_Ext_ decreased with the progression of movement, whereas CON_Ext_ and ECC_Ext_ initially decreased and either levelled off or increased in the second half of movement. Scapular muscle activity of unloaded shoulder flexion and extension changed under maximum load showing increased activity levels and an altered pattern over the course of movement.

## Introduction

For functional shoulder movements especially overhead, well-coordinated scapular motion is essential (Inman et al. 1944; Kibler 1998; Ludewig and Reynolds 2009). Previous investigations could show that the scapular motion in individuals with shoulder complaints is frequently altered (Matias and Pascoal 2006; Lin et al. 2011; Struyf et al. 2011; Kibler et al. 2013; Lawrence et al. 2014). Impaired neuromuscular activity, muscle weakness and strength imbalance of scapular stabilizing and rotating muscles are closely associated with scapular alterations as well described as scapular dyskinesis (Cools et al. 2002, 2004, 2007; Diederichsen et al. 2009; Kibler and Sciascia 2010; Phadke and Ludewig 2013; Clarsen et al. 2014). During arm elevation muscle activity levels of scapular muscles differ between individuals with shoulder pathologies like shoulder impingement and healthy controls. Studies revealed that muscle activity of the lower trapezius and serratus anterior decreased whereas muscle activity of the upper trapezius is increased during the movement of the effected shoulder (Ludewig and Cook 2000; Cools et al. 2004, 2007; Diederichsen et al. 2009; Struyf et al. 2014). Even though not all studies were able to show differences in each of the scapular stabilizing muscles, detected differences across investigations are mainly showing the same direction of alterations. The positioning of the arm and the applied load play a decisive role for the level of scapular muscle activity (Ludewig and Cook 2000). Ludewig and Cook (2000) could show that differences in scapular motion and scapular muscle activity of individuals with shoulder impingement occurred only in certain movement phases during humeral elevation with a load applied. Under loading conditions they revealed a decreased upward rotation at 60°, decreased posterior tipping at 120° of humeral elevation and in all phases an increased internal rotation. Further increased activity levels for the upper and lower trapezius were found between 60° and 120° in impingement patients. Activity alterations of the upper trapezius occurred only under submaximal loads (Ludewig and Cook 2000). A study by Lopes et al. (2015) investigated as well scapular muscle activity pattern during arm elevation in the frontal plane in subjects with and without shoulder impingement. They showed an interaction effect between group and arm elevation with increased upper trapezius activity between 30° and 60° elevation for the impingement group. However, no additional external load was applied. Huang et al. (2015) found as well an interaction effect for the upper trapezius. Individuals with impingement syndrome showed under a loaded condition higher activity levels when the arm was lowered from maximum flexion to 120° shoulder flexion. Lower trapezius and serratus anterior activity was decreased for that group without any effect of the movement phase (Huang et al. 2015). In contrast, Diederichsen et al. (2009) found increased upper trapezius activity and decreased serratus anterior activity for symptomatic individuals, but did not see any effect of movement phase on muscle activity. While some studies found an altered scapular muscle activity under unloaded and submaximal loaded conditions in impingement patients, it is still unclear to which extent these differences are evident under maximum load and if they occur in equivalent phases of arm movement. This has to be emphasized since higher loads and repetitive overhead movements put the shoulder at risk for complaints in athletes and employees who have to work overhead (Hagberg and Wegman 1987; Lo et al. 1990; McMaster and Troup 1993; Bernard 1997; Pluim et al. 2006). Even in healthy individuals, it is not clear how maximum loading of the arm affects the scapular muscle activity pattern during arm elevation and lowering. But to be able to differentiate between ‘normal and pathologic’ activity pattern, thorough investigations of scapular muscle activity in asymptomatic healthy individuals under various conditions has to be conducted first. Under unloaded passive and active arm movements, previous studies revealed that scapular muscle activity continuously increases with the progression of arm elevation and decreases again during arm lowering (Ebaugh et al. 2005; Faria et al. 2008; Ebaugh and Spinelli 2010) which highlights the increasing demands of scapular rotation and stabilization, the further the arm gets moved overhead even though no additional load is applied. It might be expected that maximum loading during shoulder flexion and extension will result in similar scapular muscle activity pattern as seen in unloaded conditions but with higher activity levels. However, so far no evidence is available to support this assumption. Therefore, this study aimed to investigate scapular muscle activity pattern during unloaded and maximal loaded isokinetic shoulder flexion and extension. Further, it will be evaluated whether differences in muscle activity are dependent on the movement phase.

## Methods

### Subjects

Fourteen recreational active volunteers (male/female: 7/7; age: 28 ± 4 years; height: 175 ± 12 cm; weight: 76 ± 16 kg, physical activity: 4 ± 2 h/week) participated in the study. All of them were pain free at the time of measurement and had neither upper limb complaints in the last six months nor any previous shoulder surgery. Each participant received an oral and written explanation of the purpose and the study design. The institutional review board approved the study. A written consent form was signed by all participants prior to participation.

### Instrumentation

**Figure 1.**
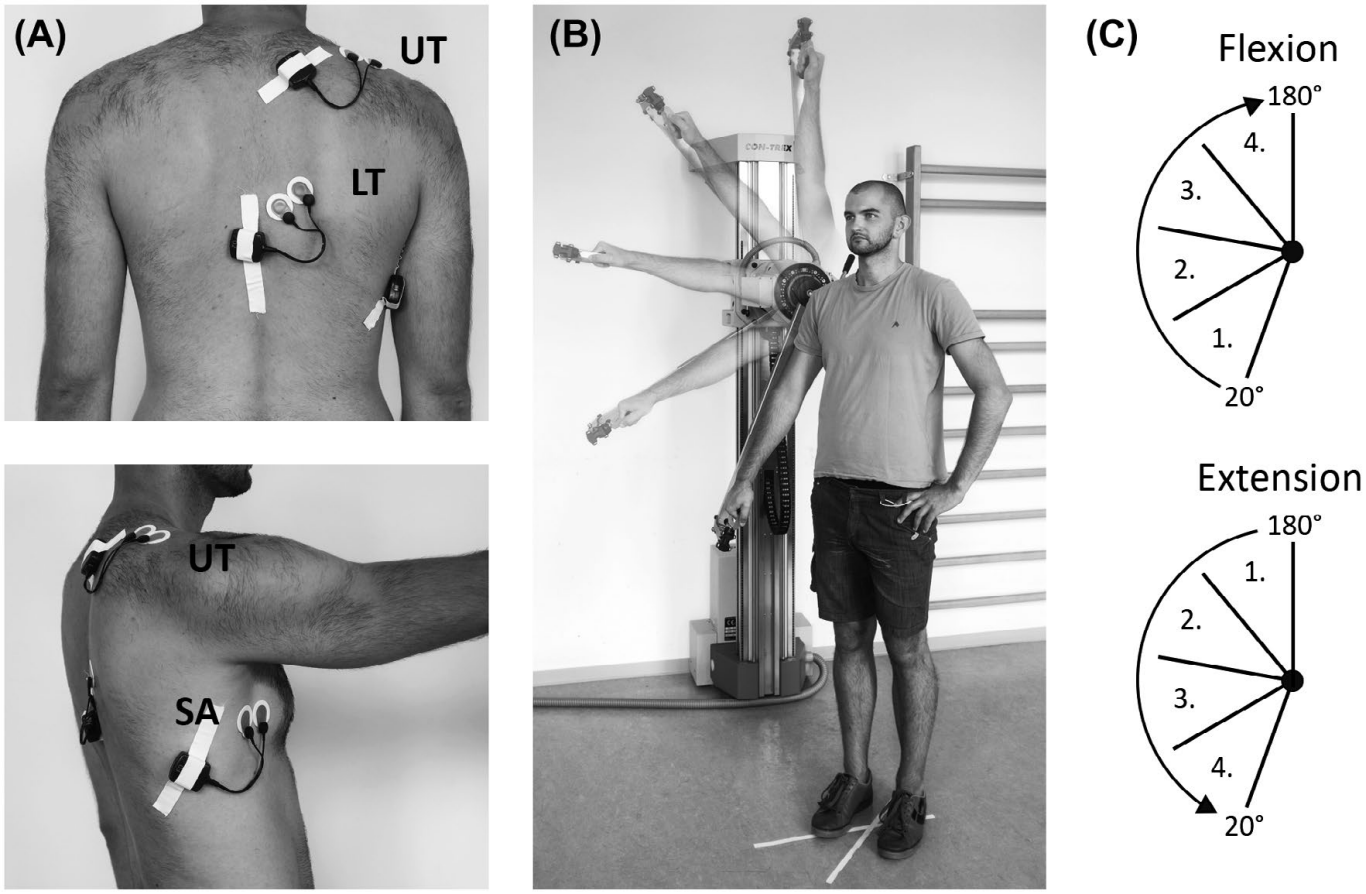
(A) EMG set-up: positioning of surface electrodes (UT = upper trapezius, LT = lower trapezius, serratus anterior). (B) Positioning of subject at dynamometer with defined ROM of 160° shoulder flexion in scapula plane. (C) Movement phases of shoulder flexion and extension.

The investigation was designed as a cross-sectional study. Participants performed shoulder flexion and extension movements in an isokinetic dynamometer (Con-Trex, WS, Physiomed Elektromedizin AG, Germany). The testing was conducted in a standing position with the participants’ right shoulder aligned to the rotational axis of the dynamometer. The hand was in a pronated position holding the handle of the adapter. Simultaneously to the isokinetic testing muscle activity of the upper trapezius (UT), lower trapezius (LT) and the serratus anterior (SA) of the right shoulder were assessed by a three-lead surface EMG (m320RX, myon AG, Switzerland). Prior to electrode placement the skin was shaved, slightly abraded and cleaned with alcohol to minimize skin impedance (<5 kΩ). All electrodes were aligned in muscle fiber direction and attached to the muscles of the right shoulder. Self-adhesive bipolar silver/silver chloride (Ag/AgCl) surface electrodes (BluSensor P, Ambu A/S, Denmark) were used with a constant inter-electrode distance of 2.0 cm. For the upper trapezius, electrodes were placed midway between the spinous process of the seventh cervical vertebra and the posterior tip of the acromion process (McQuade et al. 1998). Electrode location for the lower trapezius was 1/3 between the spinous process of the seventh thoracic vertebrae and the medial border of the scapula at the intersection of the scapula spine (McQuade et al. 1998). Electrodes for the serratus anterior muscle were placed over the seventh intercostal space, just anterior to the fibers of the latissimus dorsi (Ekstrom et al. 2004) (Figure 1(A)). The EMG signal was recorded with a 4000 Hz sampling rate. Wireless transmitters which were connected to each electrode pair amplified and digitized the signal and further transmitted it to a receiver connected to a PC.

### Procedure

Participants performed isokinetic shoulder flexion and extension at a velocity of 60°/s during a continuous passive motion (CPM), as well as during concentric (CON) and eccentric (ECC) loading. Testing in CPM was characterized by an isokinetic guided motion requiring no effort from the participant, whereas CON and ECC were performed with maximum effort. Shoulder flexion and extension were executed in a continuous alternating manner between 20° and 180° of shoulder flexion in the scapular plane. The range of motion (ROM) was predefined by the dynamometer. Prior to the measurements, a warm up of 15 repetitions of shoulder flexion and extension with submaximal and maximal repetitions were performed. Participants executed five repetitions for each isokinetic condition with a familiarization trial before each testing. Each measurement trial of five repetitions was separated by a 1-min break.

### Data analysis

Isokinetic torque was quantified by an average of the three highest peak torque values (mean ± SD; Nm). For the analysis of activity levels over the course of shoulder flexion and extension movements, the whole ROM (160°, 20–180° flexion) was divided into four equidistant movement phases of 40° increments. The phases were defined with respect to the direction and progression of the movement. For shoulder flexion, the first movement phase began at 20° and for shoulder extension at 180° flexion, respectively (Figure 1(B) and (C)). In a first step, all raw EMG data were processed with custom build software (IMAGO Process Master, LabView® based, pfitec AG, Endingen, Germany) where the signal was set to zero and full wave rectified. EMG amplitudes were analyzed by Root Mean Square (RMS) and quantified in mV. Muscle activity of single movement phases was normalized to the activity level averaged over the whole ROM during shoulder flexion and extension for each of the three scapular muscles. EMG amplitudes of single movement phases are presented as percentage of the averaged muscle activity of the whole ROM (RMS%).

The reproducibility of the measurement set-up was previously assessed and showed fair to good reliability under maximum loading conditions. Measurement variability did not exceed magnitudes of altered scapular muscle activity reported in previous studies (Wochatz et al. 2017).

### Statistical analysis

In a first step, all raw and normalized data were analyzed descriptively (mean ± SD). A two-way repeated measures analysis of variance (ANOVA, *α* = 0.05) was performed for the characterization of peak torque outputs and absolute EMG amplitudes of the three scapular muscles in regard to the isokinetic condition (CPM, CON and ECC) and the movement direction (flexion and extension). Single comparisons between conditions in relation to the movement direction were tested by paired *t*-test with a corrected significance level of *α* = 0.017. A two-way repeated measure ANOVA was as well conducted to evaluate differences of scapular muscle activity levels within each movement phase between unloaded (CPM), maximum CON and maximum ECC condition of isokinetic shoulder flexion and extension. Thereby, statistical tests were performed for each scapular muscle (UT, LT and SA) separately. In case of an interaction effect between condition and phase, *t*-tests were used for pairwise comparisons. A Bonferroni correction was performed to control for multiple testing with the significance level set to *α* = 0.013.

## Results

### Whole ROM torque and muscle activity characteristics

Peak torque values differed statistically significant (*p* < 0.05) between shoulder flexion and extension except for CPM. Also, peak torque values differed between the three isokinetic testing conditions, with CPM showing the lowest and ECC the highest outputs for both flexion and extension (Table 1).

**Table 1.** Peak torque values (mean ± SD, Nm) of shoulder flexion and extension during isokinetic testing conditions (CPM, CON, and ECC).

	CPM	CON	ECC
Flexion	3.3 ± 2.8	48.7 ± 18.8	57.3 ± 23.7
Extension	3.1 ± 6.2	61.9 ± 33.0	73.9 ± 40.7

Notes:

Isokinetic testing conditions: CPM: continuous passive motion, CON: concentric at 60°/s, ECC: eccentric at 60°/s. Statistical significant differences (*p* < 0.017) between all isokinetic conditions and the movement direction except for CPM.

**Figure 2.**
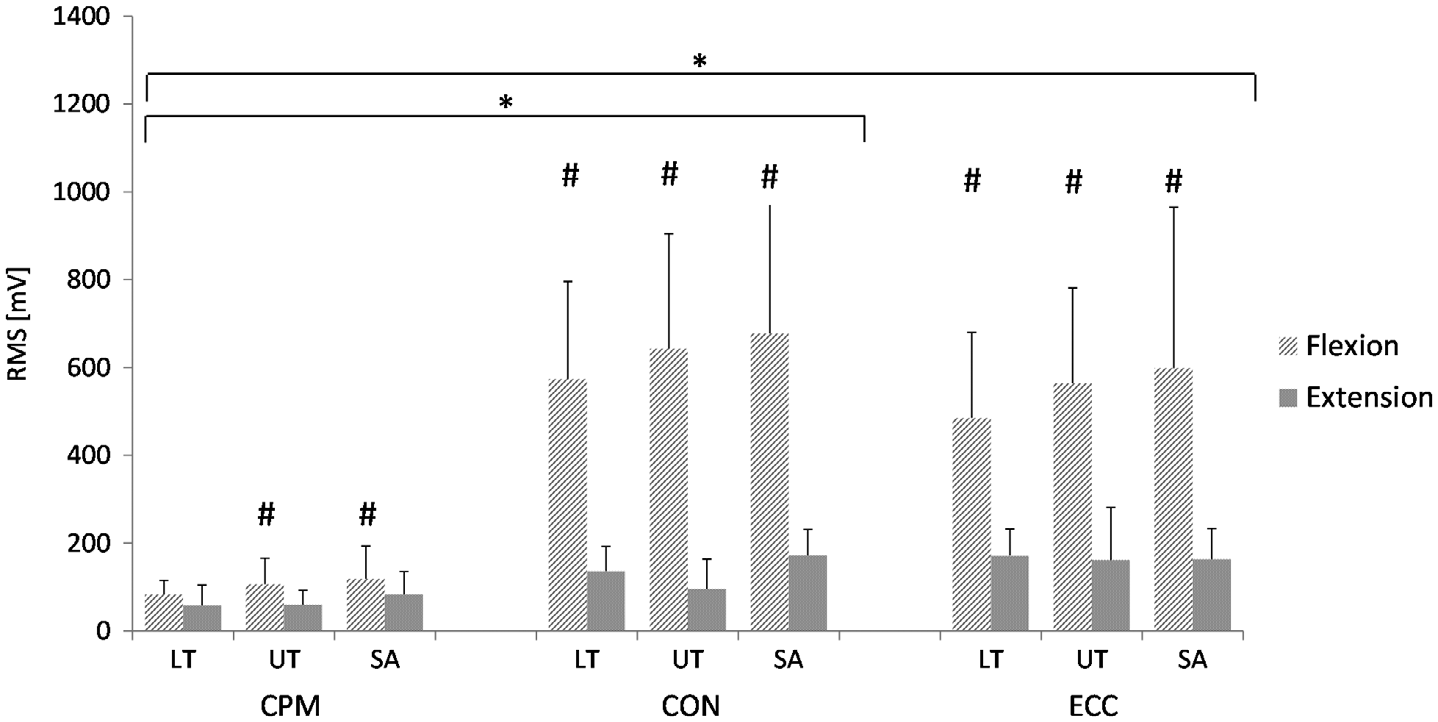
Absolute EMG amplitudes of the whole ROM for scapular muscles during isokinetic conditions of shoulder flexion and extension. Muscles: LT: Lower trapezius, UT: Upper trapezius, SA: Serratus anterior; Isokinetic testing conditions: CPM: continuous passive motion, CON: concentric at 60°/s, ECC: eccentric at 60°/s. ‘*’ indicates statistical significant differences (*p* < 0.017) in muscle activity during the loading conditions (CPM vs. CON, CPM vs. ECC). ‘#’ indicates statistical significant differences (*p* < 0.017) in muscle activity between shoulder flexion and extension.

Absolute muscle activity over the whole ROM was dependent on the loading condition and the movement direction (Figure 2). Activity levels were significantly higher during shoulder flexion than during extension, except for LT in CPM. Lowest absolute activity levels were shown during CPM for both shoulder flexion and extension, whereas no statistical significant differences in activity levels occurred during CON and ECC.

### Influence of loading condition and movement phase

**Figure 3.**
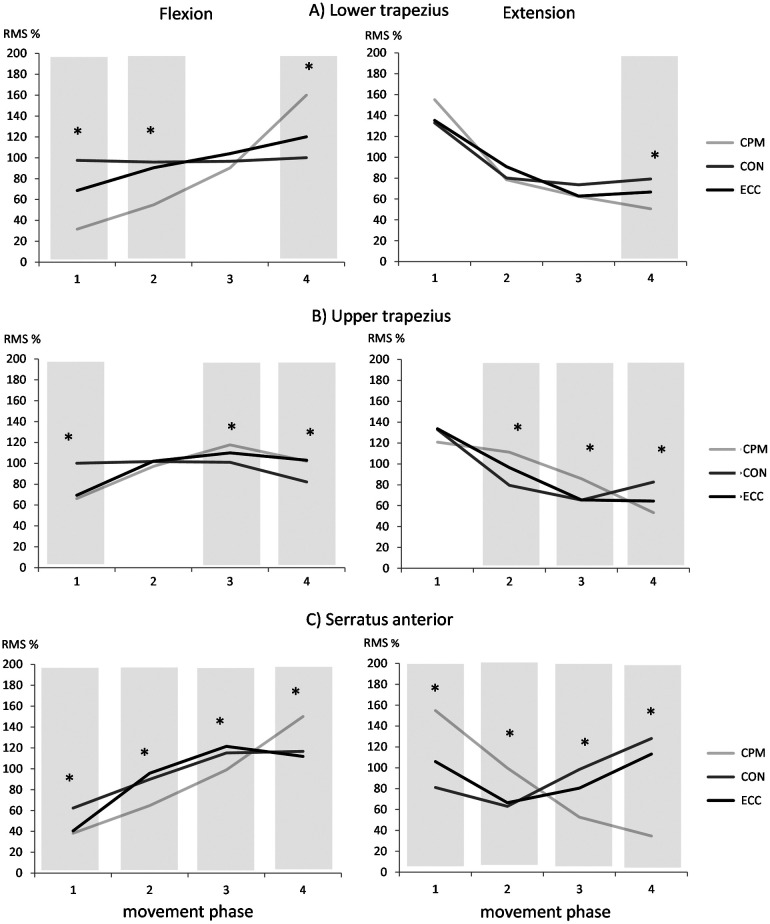
(A–C): Normalized scapular EMG activity over the course (1–4 phase) of shoulder flexion and extension of isokinetic testing conditions. CPM: continuous passive motion, CON: concentric at 60°/s, ECC: eccentric at 60°/s; Asterisk (*) indicates significant differences between isokinetic conditions (paired *t*-test, *p* = 0.013).

The ANOVA showed an interaction effect of condition and phase and revealed that muscle activity pattern of the three scapular muscles are influenced by the movement direction, the isokinetic condition and the movement phase (Table 2). Differences were apparent between unloaded (CPM) and loaded (CON and ECC) conditions as well as between the two loaded conditions CON and ECC. Statistical significant differences occurred only in specific movement phases depending on the muscle. No systematic activity pattern was apparent across muscles. The progression of muscle activity levels over the course of shoulder flexion and extension in regard to the loading condition is described below and visualized in Figure 3.

**Table 2.**
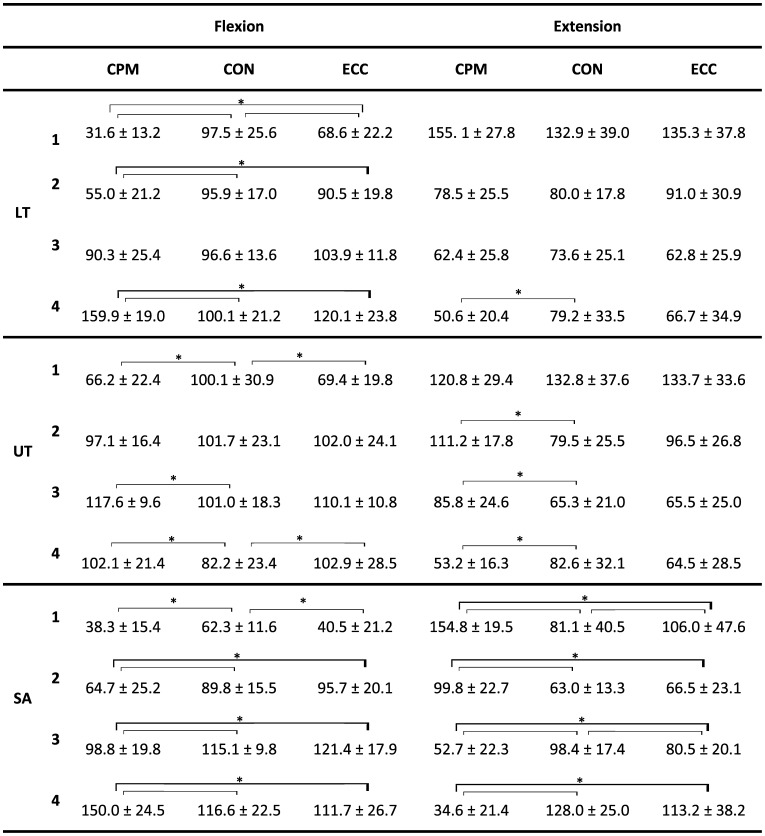
Normalized EMG activity of scapular muscles (mean ± SD) of movement phases (1–4) during shoulder flexion and extension of different isokinetic testing conditions (CPM, CON, and ECC).

Notes:

Muscles: LT = Lower trapezius, UT = Upper trapezius, SA = Serratus anterior; Isokinetic testing conditions: CPM = continuous passive motion, CON = concentric at 60°/s, ECC = eccentric at 60°/s; Asterisk (*) indicates significant differences between isokinetic conditions (paired *t*-test, *p* = 0.013).

### Activity pattern during flexion

During CPM activity levels of UT, LT and SA continuously increased by 50, 130 and 110% within the progression of shoulder flexion. Only UT showed a decrease of 15% in the final phase. During CON, activity levels of UT and LT stayed the same over the course of the movement and decreased only for UT in the final phase by 20%. Activity of SA increased over the course of the movement by 50% but leveled off in the final phase. During ECC muscle activity of UT, LT and SA initially increased by 40, 50 and 80% within the first three phases, respectively, but activity of UT and SA reached a plateau in the final phase.

### Activity pattern during extension

During CPM activity levels of UT, LT and SA continuously decreased by 70, 105 and 120%. The same pattern was seen in the first half of the movement during the loaded conditions with a decrease of up to 70% for UT and LT. Activity of both muscles levelled off in the final phase independent of their type of work (CON or ECC). During CON and ECC muscle activity of SA initially decreased by 20 and 40% but increased again by 65 and 50% in the second half of the movement reaching higher activity levels than during the beginning of shoulder extension.

## Discussion

Several studies investigated scapular muscle activity in patients with shoulder impingement syndrome and reported altered activity patterns during overhead tasks with respect to movement phase and load. Those studies were limited to passive or submaximal loaded conditions. Investigations focusing on scapular muscle demands under maximum loading are even lacking in healthy individuals. However, especially in athletes repetitive shoulder movements under maximum load are associated to pathologic changes at the shoulder. Therefore, it was the purpose of the present study to investigate scapular muscle activity pattern during unloaded and maximal loaded isokinetic shoulder flexion and extension (CON and ECC). Further, it was evaluated whether differences in muscle activity are dependent on the movement phase.

The different isokinetic testing conditions created distinct loading situations during shoulder flexion and extension. According to the loading condition, absolute muscle activity over the whole ROM was significantly lower during unloaded in comparison to the maximal loaded movements. But differences of muscle activity occurred only in certain movement phases and were dependent on the movement direction and the scapular muscle.

This investigation could show that scapular muscle activity pattern during shoulder flexion and extension alters from unloaded to maximal loaded conditions and as well between contraction types. As previous studies already described, muscle activity increases during the progression of arm elevation and decreases during the lowering of the arm (Bagg and Forrest 1986; Ebaugh et al. 2005; Faria et al. 2008; Ebaugh and Spinelli 2010). The same pattern was observed during shoulder flexion and extension of CPM condition. Only the upper trapezius reached a plateau by the end of shoulder flexion and started as well with a plateau during shoulder extension. This effect was as well seen in the investigation of Ebaugh and Spinelli (2010). Contrarily, scapular muscle activity pattern during maximum shoulder flexion and extension changed for each individual muscle. Previous investigations under submaximal load conditions showed activation pattern rather comparable to CPM than to maximal loaded conditions. Diederichsen et al. (2009) investigated scapular muscle activity pattern in individuals with and without shoulder impingement syndrome during shoulder abduction with a load of 10% of maximum voluntary isometric contractions (MVIC). They found an ongoing increase of muscle activity for LT and SA. The activity of UT increased with the course of arm elevation but decreased again in the final movement phase (Diederichsen et al. 2009). Similar activity patterns were investigated in the study by Ludewig and Cook (2000) which evaluated as well scapular muscle activity levels in individuals with and without shoulder impingement syndrome under different load conditions (unloaded, 2.3, 4.6 kg). They showed a higher plateau of activity with increasing load starting in the midrange of arm elevation. Unfortunately, these studies only investigated the elevation of the arm and therefore miss information regarding activity pattern during arm lowering. Huang et al. (2015) on the other hand, displayed only muscle activity of the lowering phase under handheld loads of 1.4 or 2.3 kg describing an initial plateau for the UT and LT which was followed by a decrease in activity. The SA showed however a constant decrease over the course of arm lowering (Huang et al. 2015). Bagg and Forrest (1988) suggest that muscle activity levels might be influenced by the relationship between the length of the muscular rotation force lever arm and the instantaneous center of rotation of the scapula based on a biomechanical model. Regarding their model, the instantaneous center of rotation is at the root of the scapular spine and moves towards the region of the acromioclavicular joint with the progression of arm elevation. The change of the rotational center alters as well the length of the rotatory force lever arm of the scapular muscles and thus influences the amount of activity. It is therefore assumed that an unfavorable mechanical position of the muscle where no rotatory force length lever arm exists results in no alterations of muscle activity. At that point, the muscle is not able to act on the segment. This explanation model seems quite suitable for results of previous studies investigating scapular muscle activity of arm elevation and lowering during unloaded or submaximal loaded conditions. It further suits as well the muscle activity pattern revealed during the CPM condition of the present study. But alterations in muscle activity levels from unloaded to maximal loaded conditions cannot be sufficiently explained by the model of Bagg and Forrest (1988). With the present results, it becomes apparent that muscular activity demands during maximum effort of arm elevation and lowering change to an extent where not only the level of the activity is increased but as well the pattern over the course of the movement. Besides the rotation of the scapula during arm elevation to allow overhead movements, scapular muscle co-contraction plays an important role to ensure a stable base of support for the development of maximum force during shoulder flexion and extension. Activity patterns seen in this study give an insight into the underlying mechanisms of scapular motion under maximum loading of overhead arm movements and therefore, might be of value for the improvement of rehabilitation and prevention programs. But for a thorough understanding of scapular demands during overhead movements under maximum effort conditions the assessment of scapular kinematics is needed to evaluate consequences of the muscle activity pattern observed in these studies. As this information is missing, it accounts for one of the limitations of the present study. Further, the isokinetic CPM mode, which was used to perform shoulder flexion and extension under unloaded conditions, moved the arm without any movement initiation of the participant. Even with several familiarization trails it could not be ruled out that participants actively moved their arm along with the device. Also, additional EMG assessment of other prime movers of shoulder flexion was not applied. Another considerable point of discussion is the classification of the four single movement phases. In accordance to the ROM of the whole movement during shoulder flexion and extension, movement phases were derived by the deviation of the whole movement into four equal phases. Visual inspections during the measurement showed variations in the humerus position which may have led to individualized movement phases and thereby added variability to muscle activity levels. Comparability to other studies might be limited as no MVIC has been conducted for the normalization. As the focus of the present study was lying on differences between movement phases of loaded and unloaded conditions, an alternative approach for the normalization was performed to overcome challenges of MVIC normalization techniques in dynamic movements (Burden and Bartlett 1999; Burden 2010). Therefore, muscle activity levels of single movement phases have been presented as a percentage of the averaged muscle activity of the whole movement. Lastly, even though application of the EMG electrodes was ensured by an experienced investigator and the use of anatomical landmarks, crosstalk may have had an influence on scapular muscle activity.

## Conclusion

Scapular muscle activity pattern of unloaded shoulder flexion and extension movements changed under maximum loading conditions. Activity levels not only increased with higher loads, but showed an altered activity pattern during the progression of shoulder flexion and extension. Differences in comparison to unloaded conditions were dependent on the scapular muscle. Whereas changes of serratus anterior activity occurred in every movement phase, changes for upper and lower trapezius were only apparent in the beginning or end phases of the movement. These findings may implicate altered demands of active scapular stabilization during the performance of overhead movements depending on the load applied. How muscle activity pattern of individuals with scapular dyskinesis, shoulder complaints or pathologies change under maximum loading conditions and whether differences to healthy individuals become more pronounced should be targeted in future investigations.

## Disclosure statement

No potential conflict of interest was reported by the authors.
